# Supplementary material for: Pyrolysis of High-Ash Natural Microalgae from Water Blooms: Effects of Acid Pretreatment
Source: Toxins (Basel). 2021 Aug 3;13(8):542. doi: 10.3390/toxins13080542 (PMC8402610; doi:10.3390/toxins13080542)
Supplement: Supplementary file 1 [file toxins-13-00542-s001.zip › toxins-1285793-supplementary.pdf]

Supplementary Materials: Pyrolysis of High-Ash Natural Microalgae from Water Blooms: Effects of Acid Pretreatment

Longfei Liu, Yichen Liu, Wenli Wang, Yue Wang, Guiying Li and Changwei Hu

Table S1. The ultimate analysis, HHV of microalgae samples.

	**NA**	**0 M**	**0.1 M**	**1 M**	**2 M**	**4 M**	**6 M**	**8 M**
C(wt%)	33.01	32.75	32.99	35.95	36.18	36.06	34.22	29.98
H(wt%)	5.14	5.24	5.26	5.28	5.31	5.25	4.98	5.84
O[a](wt%)	55.26	55.1	54.95	51.09	50.91	51.09	53.65	59.65
N(wt%)	6.59	6.91	6.8	7.68	7.6	7.6	7.15	4.53
H/C(molar ratio)	0.16	0.16	0.16	0.15	0.15	0.15	0.15	0.19
O/C(molar ratio)	1.67	1.68	1.67	1.42	1.41	1.42	1.57	1.99
N/C(molar ratio)	0.20	0.21	0.21	0.21	0.21	0.21	0.21	0.15
HHV[b](MJ/kg)	14.90	14.82	14.86	15.80	15.84	15.83	15.37	13.37

[a] by difference [b] HHV = $(3.55C^2 - 232C - 2230H + 51.2C \times H + 131N + 20600) \times 10^{-3}$.

Table S2. The HHV of bio-oil from different samples by pyrolysis.

	**NA**	**0 M**	**0.1 M**	**1 M**	**2 M**	**4 M**	**6 M**	**8 M**
C(wt%)	61.43	63.56	63.88	63.51	64.14	63.34	63.54	62.22
H(wt%)	8.06	8.27	8.27	8.33	8.32	8.28	8.51	8.24
O[a](wt%)	20.39	17.3	16.68	17.44	17.26	17.93	17.75	19.19
N(wt%)	10.12	10.87	11.17	10.72	10.28	10.45	10.20	10.35
H/C(molar ratio)	0.13	0.13	0.13	0.13	0.13	0.13	0.13	0.13
O/C(molar ratio)	0.33	0.27	0.26	0.27	0.27	0.28	0.28	0.31
N/C(molar ratio)	0.16	0.17	0.17	0.17	0.16	0.16	0.16	0.17
HHV[b](MJ/kg)	28.45	30.09	30.34	30.10	30.44	29.90	30.24	29.14

[a] by difference [b] HHV = $(3.55C^2 - 232C - 2230H + 51.2C \times H + 131N + 20600) \times 10^{-3}$.

Table S3. The relative content of small molecular compounds in the bio-oil.

	Ash	0 M	0.1 M	1 M	2 M	4 M	6 M	8 M
**Hydrocarbons**	**12.90%**	**15.60%**	**15.66%**	**19.86%**	**26.67%**	**19.95%**	**18.17%**	**15.42%**
Heptadecane	9.68%	11.38%	10.19%	12.75%	16.29%	11.82%	10.82%	8.59%
Heptadecane,9-hexyl-	3.22%	4.22%	3.38%	4.34%	7.40%	5.40%	4.34%	5.08%
dodecane,5,8-diethyl-	—	—	2.09%	2.77%	2.99%	2.72%	3.01%	1.75%
**Aromatic Compounds**	**6.31%**	**12.66%**	**13.07%**	**3.14%**	**1.97%**	**0.54%**	**2.07%**	**0.52%**
benzene,1-isocyano-2-methyl-	2.97%	9.01%	7.59%	2.10%	1.97%	0.54%	2.07%	0.52%
Carbamic acid, methyl-, phenylester	0.83%	1.60%	1.23%	—	—	—	—	—
phenol,3-methyl-	2.51%	2.06%	2.22%	—	—	—	—	—
benzyloxymethylimine	—	—	2.03%	1.04%	—	—	—	—
**Carboxylic acids**	**41.14%**	**29.41%**	**34.29%**	**48.52%**	**51.62%**	**62.53%**	**59.83%**	**63.18%**
n-hexadecanoic acid	22.44%	19.12%	26.21%	41.88%	45.42%	58.77%	56.49%	54.95%
dodecanoic acid, 3-hydroxy-	2.03%	3.11%	1.00%	2.04%	0.85%	1.67%	1.18%	0.73%
9-hexadecenoic acid	1.73%	3.10%	1.90%	4.60%	5.35%	2.09%	2.16%	2.86%
acetic acid	5.92%	1.52%	2.22%	—	—	—	—	—
propanoic acid, 2-methyl-	1.33%	—	—	—	—	—	—	—
butanoic acid	3.19%	—	—	—	—	—	—	—
pentanoic acid	1.91%	—	—	—	—	—	—	—
Pentadecanoic acid,14-methyl-, methylester	2.60%	2.56%	2.96%	—	—	—	—	—
E-9-tetradecenoic acid	—	—	—	—	—	—	—	1.28%
Oleic acid	—	—	—	—	—	—	—	3.36%
**Nitrogen Compounds**	**29.10%**	**31.48%**	**27.42%**	**17.11%**	**12.20%**	**10.00%**	**9.80%**	**18.52%**
hexadecanamide	11.31%	16.22%	11.91%	11.67%	7.97%	7.27%	7.85%	12.05%
hexadecanenitrile	10.40%	10.11%	8.85%	3.02%	3.53%	2.73%	1.95%	6.47%
hydrazine,methyl-	—	—	—	0.38%	0.70%	—	—	—
pyrrole	0.93%	1.01%	1.56%	—	—	—	—	—
pentanenitrile,4-methyl-	—	0.99%	0.73%	1.30%	—	—	—	—
1H-pyrrole,3-methyl-	0.77%	0.47%	0.97%	—	—	—	—	—
1H-pyrrole,2-methyl-	0.47%	0.38%	1.04%	—	—	—	—	—
1H-pyrrole,2-ethyl-4-methyl-	0.38%	—	—	—	—	—	—	—
pyrrole,4-ethyl-2-methyl-	0.42%	—	—	—	—	—	—	—
formide, N-methyl-N-4-	2.85%	2.30%	2.36%	0.73%	—	—	—	—
2-[2-methyl-propenyl]-cyclohexanone oxime	1.57%	—	—	—	—	—	—	—
**Oxygen Compounds**	**10.55%**	**10.85%**	**9.56%**	**11.38%**	**7.54%**	**6.98%**	**10.14%**	**2.35%**
Z-9-pentadecenol	3.03%	7.33%	4.63%	6.88%	4.28%	3.90%	6.69%	—
13-heptadecyn-1-ol	1.36%	2.28%	2.54%	1.83%	3.26%	3.08%	3.45%	2.35%
cyclohexanone,4-hydroxy-	0.93%	—	—	—	—	—	—	—
2-cyclopenten-1-one,2-methyl-	1.19%	1.23%	—	—	—	—	—	—
1,2,3,4-cyclopentaneterol	1.01%	—	—	—	—	—	—	—
pyrrolidone,5-[3-methoxy-butyl]-	2.25%	—	—	—	—	—	—	—
corymbolone	0.77%	—	—	—	—	—	—	—
7-hexadecyn-1-ol	—	—	1.49%	1.47%	—	—	—	—
2-hexadecanol	—	—	0.90%	1.19%	—	—	—	—

Table S4. Joint hypotheses test between acid concentration and variations.

Variations	Levene statistic	df1	df2	Significance
Ash content	0.366	6	14	0.889
Ca	3.927	6	14	0.016
Mg	0.800	6	14	0.586
Al	1.005	6	14	0.460
Carbohydrate content	0.442	6	14	0.839
Liquid yield	0.520	6	14	0.784

Table S5. ANOVA analysis between acid concentration and variations.

Variations	F value	Significance
Ash content	822.434	0.000
Ca	487.785	0.000
Mg	128.118	0.000
Al	14,680.731	0.000
Carbohydrate content	80.393	0.000
Liquid yield	108.339	0.000

Table S6. Post hoc test of ANOVA analysis between acid concentration and ash content.

	Acid Concentration	N	α = 0.05 Subsets				
			1	2	3	4	5
Student-Newman-Keuls [a]	2.0	3	21.1				
	1.0	3		28.6			
	4.0	3			30.9		
	6.0	3				32.9	
	0.1	3				33.6	
	0.0	3				33.7	
	8.0	3					43.3
	Significance		1.000	1.000	1.000	0.069	1.000

The group mean of a subset of the same class was displayed. [a] The harmonic mean sample size = 3.000.

Table S7. Post hoc test of ANOVA analysis between acid concentration and content of Ca.

	Acid Concentration	N	α = 0.05 Subsets				
			1	2	3	4	5
Student-Newman-Keuls [a]	2.0	3	0.32				
	1.0	3	0.44				
	0.1	3		1.05			
	0.0	3			2.10		
	4.0	3			2.32		
	6.0	3				4.83	
	8.0	3					5.42
	Significance		0.375	1.000	0.115	1.000	1.000

The group mean of a subset of the same class was displayed. [a] The harmonic mean sample size = 3.000.

Table S8. Post hoc test of ANOVA analysis between acid concentration and content of Mg.

	Acid Concentration	N	α = 0.05 Subsets			
			1	2	3	4
Student-Newman-Keuls [a]	2.0	3	0.50			
	4.0	3	0.52			
	8.0	3		1.07		
	6.0	3			1.18	
	0.1	3			1.21	1.21
	0.0	3				1.30
	1.0	3				1.31
	Significance		0.657	1.000	0.507	0.094

The group mean of a subset of the same class was displayed. [a] The harmonic mean sample size = 3.000.

Table S9. Post hoc test of ANOVA analysis between acid concentration and content of Al.

	Acid Concentration	N	α = 0.05 Subsets						
			1	2	3	4	5	6	7
Student-Newman-Keuls [a]	4.0	3	5.78						
	2.0	3		6.27					
	6.0	3			14.15				
	8.0	3				14.64			
	1.0	3					17.76		
	0.1	3						21.95	
	0.0	3							23.32
	Significance		1.000	1.000	1.000	1.000	1.000	1.000	1.000

The group mean of a subset of the same class was displayed. [a] The harmonic mean sample size = 3.000.

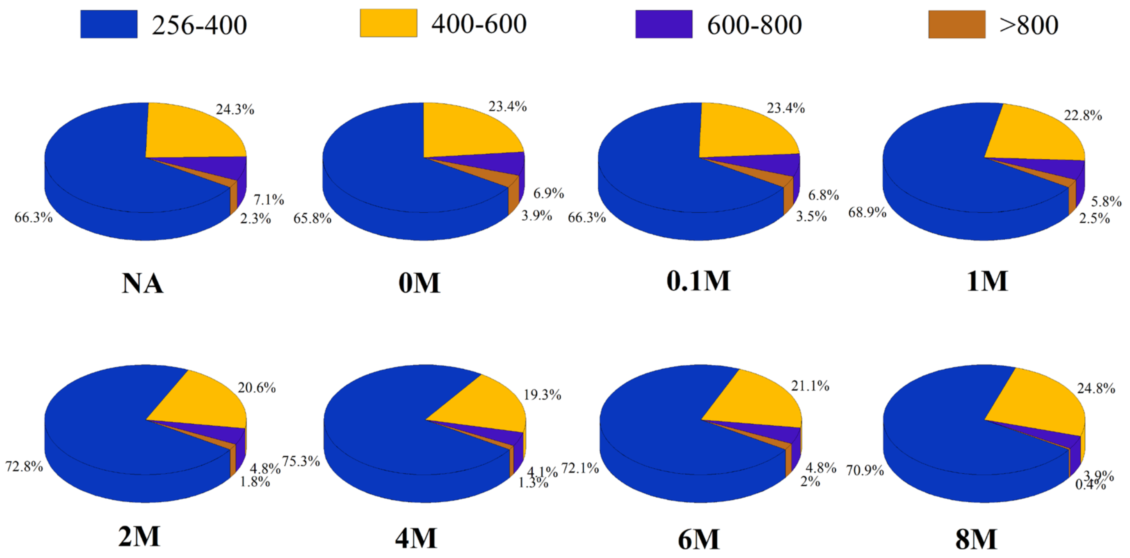

Figure S1. The molecular weight distribution of the bio-oil.
